# Using amino acid features to identify the pathogenicity of influenza B virus

**DOI:** 10.1186/s40249-022-00974-0

**Published:** 2022-05-04

**Authors:** Zheng Kou, Xinyue Fan, Junjie Li, Zehui Shao, Xiaoli Qiang

**Affiliations:** 1 Institute of Computing Science and Technology, Guangzhou University, Guangzhou, 510006 China; 2 School of Computer Science and Cyber Engineering, Guangzhou University, Guangzhou, 510006 China

**Keywords:** Influenza B virus, Pathogenicity, Amino acid feature, Machine learning

## Abstract

**Background:**

Influenza B virus can cause epidemics with high pathogenicity, so it poses a serious threat to public health. A feature representation algorithm is proposed in this paper to identify the pathogenicity phenotype of influenza B virus.

**Methods:**

The dataset included all 11 influenza virus proteins encoded in eight genome segments of 1724 strains. Two types of features were hierarchically used to build the prediction model. Amino acid features were directly delivered from 67 feature descriptors and input into the random forest classifier to output informative features about the class label and probabilistic prediction. The sequential forward search strategy was used to optimize the informative features. The final features for each strain had low dimensions and included knowledge from different perspectives, which were used to build the machine learning model for pathogenicity identification.

**Results:**

The 40 signature positions were achieved by entropy screening. Mutations at position 135 of the hemagglutinin protein had the highest entropy value (1.06). After the informative features were directly generated from the 67 random forest models, the dimensions for class and probabilistic features were optimized as 4 and 3, respectively. The optimal class features had a maximum accuracy of 94.2% and a maximum Matthews correlation coefficient of 88.4%, while the optimal probabilistic features had a maximum accuracy of 94.1% and a maximum Matthews correlation coefficient of 88.2%. The optimized features outperformed the original informative features and amino acid features from individual descriptors. The sequential forward search strategy had better performance than the classical ensemble method.

**Conclusions:**

The optimized informative features had the best performance and were used to build a predictive model so as to identify the phenotype of influenza B virus with high pathogenicity and provide early risk warning for disease control.

**Graphical Abstract:**

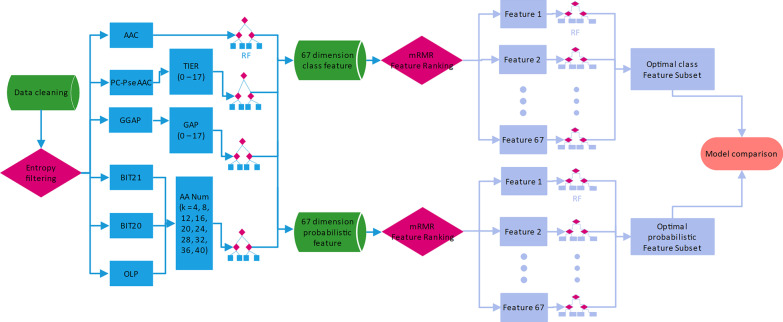

**Supplementary Information:**

The online version contains supplementary material available at 10.1186/s40249-022-00974-0.

## Background

Influenza B virus (IBV) belongs to the Orthomyxoviridae family, and its genome is composed of eight negative-strand RNA of different lengths [1, 2]. As a pathogen that can cause human respiratory diseases, IBV was first isolated from clinical samples in 1940 [3]. According to the antigen characteristics of the hemagglutinin protein, two lineages of IBV were reported: Victoria-like virus and Yamagata-like virus [4]. IBV can cause local outbreaks or seasonal epidemics with a high mortality rate in children and adolescents, so it poses a serious threat to public health [5–10].

There are at least 11 viral proteins encoded in the genome of IBV: polymerase basic protein 2 (PB2), polymerase basic protein 1 (PB1), polymerase acid protein (PA), hemagglutinin (HA), nucleoprotein (NP), neuraminidase (NA), glycoprotein (NB), matrix protein (M), matrix protein 2 (BM2), nonstructural protein 1 (NS1), and nuclear export protein (NEP) [11]. The pathogenicity of influenza viruses to mammals is determined by amino acid mutation. For example, mutations in PB2 increase the virulence for influenza A virus isolated from avian species and swine [12, 13]. The screening of the key amino acid mutation is crucial for understanding the pathogenicity of IBV, which can be used to evaluate its virulence and predict even pandemic risk. Although several mutations are related to viral pathogenicity, comprehensive screening has not been achieved [14–17]. System identification of amino acid mutations is expected with the increase of genome data for IBV [18–22].

The pathogenicity of any influenza virus is an important indicator for pandemic risk. Computational tools in the field of machine learning have been used to identify phenotype of biological data [23, 24]. Machine learning techniques gain knowledge from viral protein sequences and represent viruses by optimal features [25]. A model with good performance evaluates the pathogenicity of IBV and predicts the ability of transmission. With the increase of genome data in the public database, machine learning methods are ideal tools for phenotype identification of IBVs [26].

To capture the key information of mutant amino acids of viral proteins, different feature encoding algorithms from multiple perspectives are considered in this paper, such as compositional information, position-specific information, and physicochemical properties. The amino acid composition (AAC) is a simple feature descriptor for sequence analysis [27]. Parallel correlation-based pseudo-amino-acid composition (PC-PseAAC) measures the parallel correlation of any two amino acids in the signature positions [28]. The standard amino acid alphabet is classified and grouped based on five physicochemical properties: polarity, secondary structure, molecular volume, codon diversity, and electrostatic charge [29]. The orthotropic one-hot and overlapping properties can be used to describe amino acids [30]. Different types of information for amino acid features can be used to construct a machine learning model with good performance.

In this paper, we propose a feature representation algorithm to identify the pathogenicity of IBV. Informative features about the class label or probabilistic prediction were learned from 67 random forest (RF) classifiers. A final predictor was proposed with the use of optimized informative features and performed impressively. Thus, we posit that the proposed method is a powerful tool for pathogenicity identification of IBVs at a large scale, which can aid in warning about transmission risk as well as benefit public health.

## Methods

### Data set

To describe the transmission dynamic of IBV, surveillance data from 1997 to 2020 were collected from the United States Centers for Disease Control and Prevention (https://www.cdc.gov/flu/weekly/fluviewinteractive.htm). Because of the impact of COVID-19, sparse data from the 2020 to 2021 and 2021 to 2022 influenza seasons were omitted. Regarding pathogenicity, the percentage of IBV in all positive samples of influenza virus per season was calculated. As the number of positive tests changes every year, the positive test rate was used to reflect the pathogenicity.

To construct a machine learning model, protein data of IBVs isolated from the US were downloaded from the GISAID public database (http://platform.gisaid.org/epi3/frontend) [31, 32]. To reduce the redundancy of sequence similarity and cover the integrity of the viral genome, the raw data were processed before modeling [18]. The clustering algorithm was used to reduce the redundancy of viral sequences. Only strains with the full length of viral proteins were considered. Ambiguous amino acid residues were checked and edited carefully. Strains with low-quality sequencing were also removed. The final dataset included all 11 influenza virus proteins (PB2, PB1, PA, HA, NP, NA, NB, M1, BM2, NS1, and NEP) of 1724 strains (see Additional file 1).

### Signature amino acid position

Viral proteins have important biological functions and play key roles during infection and transmission. The total length of the 11 viral proteins was 4708 amino acids. Although fast mutation rates have been observed, most amino acid residues in the 11 viral proteins were conserved. Signature positions were screened to reduce the computing complexity. Entropies in each position of the 11 viral proteins were calculated and measured with ${E}_{i}= -\sum_{j=1}^{21}{P}_{i,j}\mathrm{log}\left({P}_{i,j}\right)$, where ${P}_{i,j}$ is the frequency of amino acid $j$ at position $i$. Deletion or insertion was also considered. High values reflect frequent mutations in any given position [33].

### Amino acid composition

To identify the pathogenicity of IBV using a machine learning method, the features for amino acids in signature positions should be encoded as input. Six different encoding algorithms from multiple perspectives, including compositional information, position-specific information, and physicochemical properties, were used in this paper. The AAC is simple descriptor for the viral protein sequence of IBV [27]. The AAC method calculates the frequency of an amino acid in signature positions. The gap (deletion or insertion) was also considered. A 21-dimensional feature vector was used to represent each strain.

### PC-PseAAC

The PC-PseAAC is an updated AAC that calculates the parallel correlation of any two amino acids in a protein or peptide sequence [28]. For each strain used in this paper, the PC-PseAAC feature vector is measured as

$$PC-PseAAC={\left[{fv}_{1},\dots ,{fv}_{21},{fv}_{21+1},\dots ,{fv}_{20+\uplambda }\right]}^{T},$$

where

$${fv}_{u}=\left\{\begin{array}{c}\frac{{f}_{u}}{{\sum }_{i=1}^{21}{f}_{i}+w{\sum }_{j=1}^{\uplambda }{\theta }_{j}}, 1\le u\le 21\\ \frac{w{\theta }_{u-21}}{{\sum }_{i=1}^{21}{f}_{i}+w{\sum }_{j=1}^{\uplambda }{\theta }_{j}}, 21+1\le u\le 21+\lambda \end{array}\right..$$

Here, $u$ is an integer that changes with $\uplambda$; ${fv}_{u}$ $(1\le u\le 21)$ represents the normalized appearance frequency of the 20 amino acids and a gap for each strain; λ represents the highest tier of the correlation along signature positions; ${\theta }_{j}$ $(j=\mathrm{1,2},\dots ,\uplambda )$ is the correlation function that measures the $j$-tier sequence-order correlation between all the $j$-th most contiguous residues along signature positions [18].

### G-gap dipeptide composition

Th G-gap dipeptide composition (GGAP) measures the dipeptide composition coupled with local order information of any two interval residues within protein sequences. GGAP is represented as

$$GGAP\left(g\right)=\left({fv}_{1}^{g},{fv}_{2}^{g},\dots ,{fv}_{441}^{g}\right),$$

where ${fv}_{i}^{g}$ is the frequency of the $i$-th ($i$= 1,2, …, 441) g-gap dipeptide in signature positions [18]. The dimension of the GGAP feature vector is 21 × 21 = 441. Deletion or insertion was also computed.

### Twenty-bit features

In addition to methods based on the frequency of the amino acid, features about position-specific information and physicochemical properties were also used. The standard 20 amino acids were grouped according to the five physicochemical properties: polarity, secondary structure, molecular volume, codon diversity, and electrostatic charge [29]. For each physicochemical property, the 20 amino acids were clustered into three groups, and deletion/insertion was regarded as the fourth group [18]. A total of 20 groups for each alphabet in the signature positions were achieved. Each residue was encoded as a 20-bit vector comprising 0/1 elements, where the position of the bit was set to 1 if the residue belonged to the corresponding group, and 0 otherwise. The signature positions in this paper were screened with the method of entropy. The top k residues with the highest values of entropy were selected, and the dimension of the feature vector was 20 × k [18].

### Twenty-one-bit features

For position-specific information of signature positions, each alphabet was encoded into a 21-bit 0/1 vector as in one-hot encoding, for example, Ala by 1,0,0,0,0,0,0,0,0,0,0,0,0,0,0,0,0,0,0,0,0 or deletion/Insertion by 0,0,0,0,0,0,0,0,0,0,0,0,0,0,0,0,0,0,0,0,1). Therefore, the top k residues were encoded with a 21 × k dimensional feature vector [18].

### Overlapping property features

Each amino acid was classified into 10 groups based on overlapping physicochemical properties [30]. The 10 physicochemical properties and their corresponding amino acid groups were as follows: Aromatic = {F, Y, W, H}, Negative = {D, E}, Positive = {K, H, R}, Polar = {N, Q, S, D, E, C, T, K, R, H, Y, W}, Hydrophobic = {A, G, C, T, I, V, L, K, H, F, Y, W, M}, Aliphatic = {I, V, L}, Tiny = {A, S, G, C}, Charged = {K, H, R, D, E}, Small = {P, N, D, T, C, A, G, S, V}, and Proline = {A, S, G, C}. Deletion/Insertion was regarded as the 11th group. The alphabet in the signature positions was then encoded by a 11-dimensional 0/1 vector. The position of the vector was set to 1 if the residue belonged to the physicochemical property and 0 otherwise. In this paper, the top k residues were encoded with a 11 × k feature vector [18].

### RF predictor

The RF algorithm was used to output the informative features about the class label and probabilistic prediction [18]. R 3.5.0 (Lucent Technologies, Jasmine Mountain, USA) was used to perform the RF algorithm, and the tree number was set to 500 by default [34].

### Framework for pathogenicity identification

**Fig. 1:**
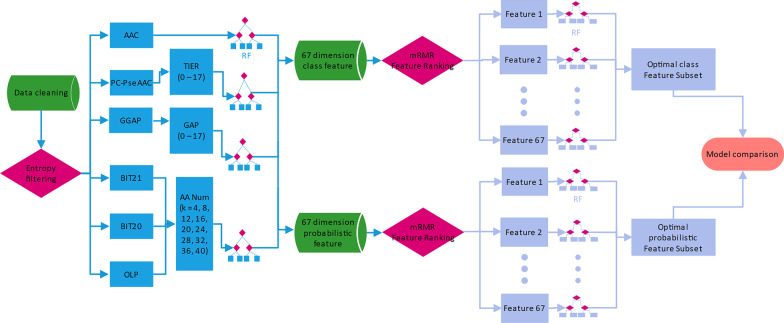
Flowchart of pathogenicity identification of IBV. The 40 signature positions based on entropy were first screened after data were downloaded and cleaned. Six encoding methods of amino acids with changeable parameters were used to extract features. Then, 67 descriptors were proposed, and two types of informative outputs from the RF method were obtained to be further optimized with the mRMR algorithm and the SFS strategy. Each strain was finally represented by two optimized informative features with the low dimension ‘class’ and ‘prob.’ These optimal subsets were used to construct predictive models

The framework for pathogenicity identification of IBV is shown in Fig. 1. Two types of features were hierarchically used to represent IBV: amino acid features and informative features [27]. Amino acid features were directly delivered from 67 feature descriptors and were input into the RF predictors. The informative features about the class label and probabilistic prediction were then generated and further optimized. The optimal subset of informative features to represent each strain had low dimensions and included knowledge from different perspectives, which were expected to improve the performance of the identification model.

The six amino acid encoding algorithms were AAC, PC-PseAAC, GGAP, 20-Bit features (BIT20), 21-bit features (BIT21), and overlapping property features (OLP). The variate k is the common parameter for BIT20, BIT21, and OLP, and controls the dimension of amino acid features. k varied from 4 to 40 by a step size of 4. The maximum was set to 40 because there were 40 signature positions. The 67 feature descriptors under different parameters were produced (Table 1). The class and probabilistic features were then provided by each RF model. The class feature is the predicted class label. The positive samples were marked as 1, and the negative samples were marked as 0. The probabilistic feature is the probability of the positive label. For each type of informative feature, the 67 values were concatenated into a new vector. Each strain was then represented by two informative features.

**Table 1:** Summary of feature descriptor and feature number

Feature descriptor	Feature type	Feature number	Feature descriptor	Feature type	Feature number
1	AAC	20	35	GGAP (g = 15)	441
2	PseAAC (λ = 0)	21	36	GGAP (g = 16)	441
3	PseAAC (λ = 1)	22	37	GGAP (g = 17)	441
4	PseAAC (λ = 2)	23	38	BIT20 (k = 4)	80
5	PseAAC (λ = 3)	24	39	BIT20 (k = 8)	160
6	PseAAC (λ = 4)	25	40	BIT20 (k = 12)	240
7	PseAAC (λ = 5)	26	41	BIT20 (k = 16)	320
8	PseAAC (λ = 6)	27	42	BIT20 (k = 20)	400
9	PseAAC (λ = 7)	28	43	BIT20 (k = 24)	480
10	PseAAC (λ = 8)	29	44	BIT20 (k = 28)	560
11	PseAAC (λ = 9)	30	45	BIT20 (k = 32)	640
12	PseAAC (λ = 10)	31	46	BIT20 (k = 36)	720
13	PseAAC (λ = 11)	32	47	BIT20 (k = 40)	800
14	PseAAC (λ = 12)	33	48	BIT21 (k = 4)	84
15	PseAAC (λ = 13)	34	49	BIT21 (k = 8)	168
16	PseAAC (λ = 14)	35	50	BIT21 (k = 12)	252
17	PseAAC (λ = 15)	36	51	BIT21 (k = 16)	336
18	PseAAC (λ = 16)	37	52	BIT21 (k = 20)	420
19	PseAAC (λ = 17)	38	53	BIT21 (k = 24)	504
20	GGAP (g = 0)	441	54	BIT21 (k = 28)	588
21	GGAP (g = 1)	441	55	BIT21 (k = 32)	672
22	GGAP (g = 2)	441	56	BIT21 (k = 36)	756
23	GGAP (g = 3)	441	57	BIT21 (k = 40)	840
24	GGAP (g = 4)	441	58	OLP (k = 4)	44
25	GGAP (g = 5)	441	59	OLP (k = 8)	88
26	GGAP (g = 6)	441	60	OLP (k = 12)	132
27	GGAP (g = 7)	441	61	OLP (k = 16)	176
28	GGAP (g = 8)	441	62	OLP (k = 20)	220
29	GGAP (g = 9)	441	63	OLP (k = 24)	264
30	GGAP (g = 10)	441	64	OLP (k = 28)	308
31	GGAP (g = 11)	441	65	OLP (k = 32)	352
32	GGAP (g = 12)	441	66	OLP (k = 36)	396
33	GGAP (g = 13)	441	67	OLP (k = 40)	440
34	GGAP (g = 14)	441			

*AAC* amino acid composition, *PC-PseAAC* parallel correlation-based pseudo-amino-acid composition, *GGAP* the G-gap dipeptide composition, *BIT20* twenty-bit feature, *BIT21* twenty-one-bit feature, *OLP* overlapping property feature

In this paper, two 67-dimensional features were further optimized to reduce computational complexity and increase performance. The minimum-redundancy maximum-relevancy (mRMR) algorithm was used to rank informative features by importance scores [35]. Moreover, the sequential forward search (SFS) strategy was used to increase the informative features from the ranked list one by one. The subset with the best performance was considered to have the optimal features and was proposed to construct the final model for pathogenicity identification [27].

### Performance evaluation

Four popular metrics for performance evaluation, Sensitivity (SN), Specificity (SP), Accuracy (ACC), and Matthews correlation coefficient (MCC), were used as follows:

$$SN=\frac{TP}{TP+FN}\times 100\%$$

$$SP=\frac{TN}{TN+FP}\times 100\%$$

$$ACC=\frac{TP+TN}{TP+TN+FP+FN}\times 100\%$$

$$MCC=\frac{TP\times TN+FP\times FN}{\sqrt{\left(TP+FN\right) \left(TP+FP\right) \left(TN+FN\right) \left(TN+FP\right)}}\times 100\%$$

where TP indicates the correct number of strains with the phenotype of high pathogenicity; TN represents the correct number of strains with the phenotype of low pathogenicity; FP indicates the wrong number of strains with the phenotype of low pathogenicity; and FN is the wrong number of strains with the phenotype of high pathogenicity.

The receiver operating characteristic (ROC) curve was also used to evaluate the overall performance [36]. The curve is generated by plotting the true positive rate (TPR) against the false positive rate (FPR) under different classification thresholds. The area under the ROC curve (AUC) was used to evaluate the predictive performance. A larger AUC value suggests that the model achieves a better performance [26].

## Results

### Pathogenicity of IBV

**Fig. 2:**
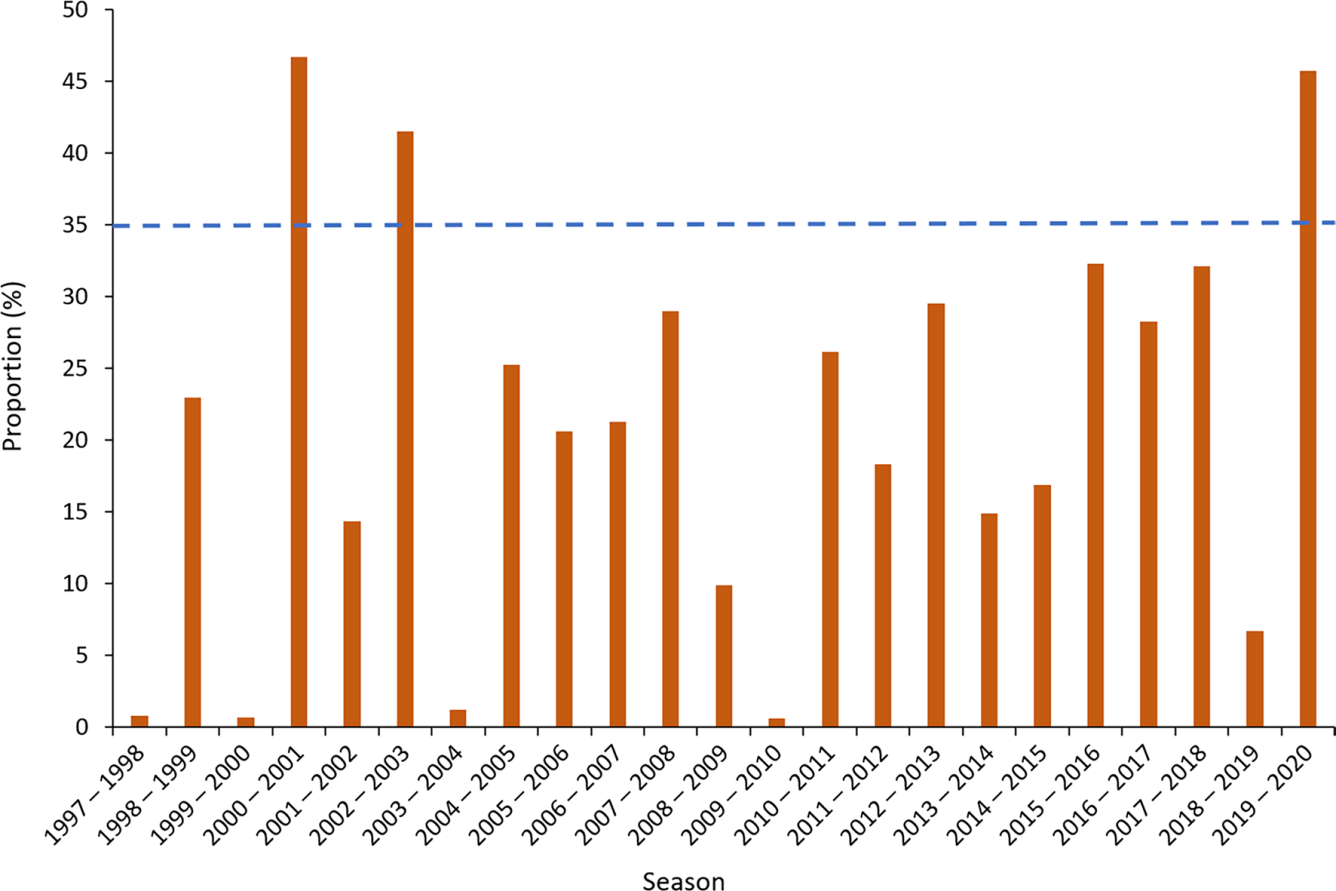
Proportion of IBV in all positive samples per influenza season. The *x*-axis represents the seasons from 1997 to 2000. The *y*-axis represents the positive proportion for IBV. The ratio of 35% is shown by the dotted blue line

To summarize the transmission dynamic of IBV, US surveillance data from 1997 to 2020 were collected. The percentage of IBV in all positive samples of human influenza virus was calculated for each influenza season. The positive rates for the 2000–2001, 2002–2003, and 2019–2020 seasons were more than 35% (Fig. 2). IBV isolated from the three screened seasons with high positive rates were regarded as positive samples, while those in the other 20 seasons had low pathogenicity and were regarded as negative samples. The final dataset for model construction was composed of 1724 strains. Two groups were classified: (1) 865 viruses (positive sample; high pathogenicity; 2000–2001, 2002–2003, 2019–2020 seasons) and 859 viruses (negative sample; low pathogenicity; other 20 seasons). The information related to these strains is summarized in Additional file 1.

### Signature position

**Fig. 3:**
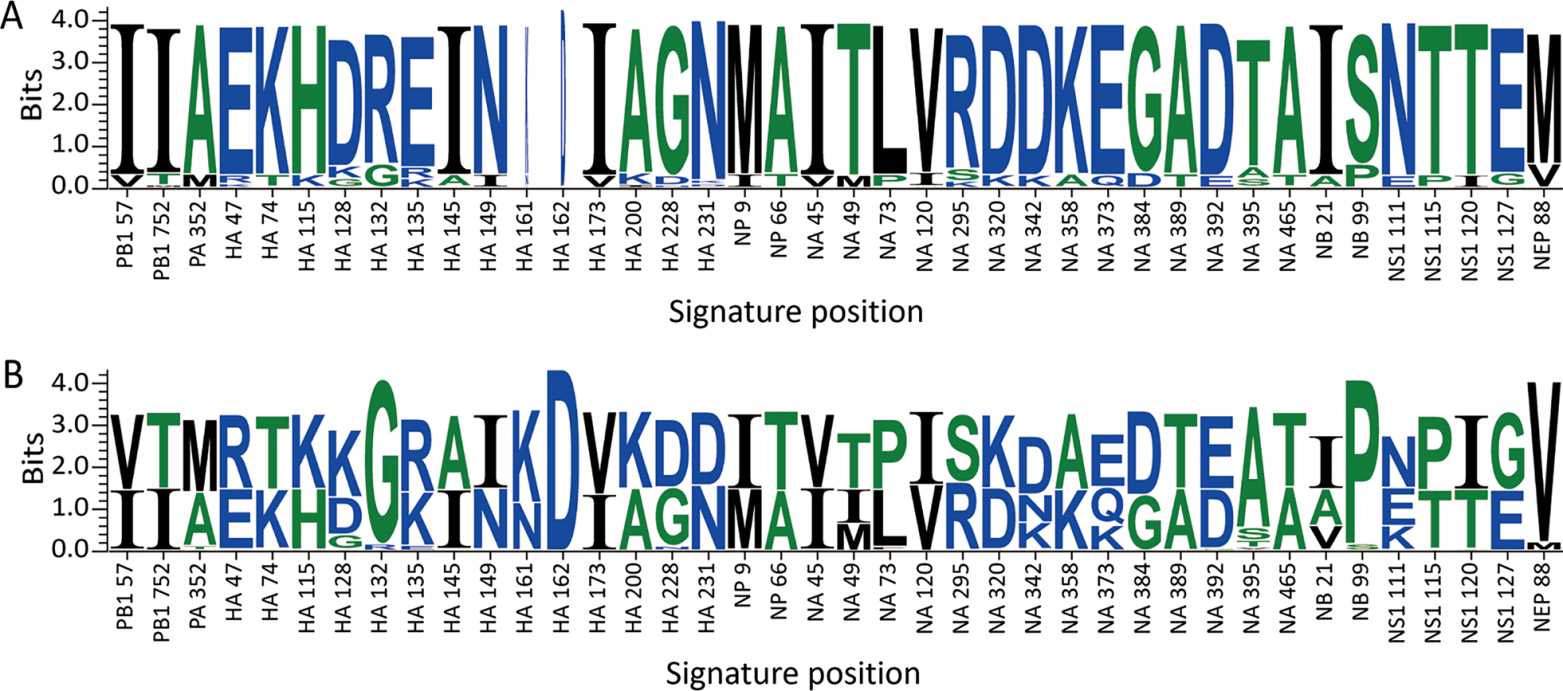
Signature positions in the 11 viral proteins. **A** Profile of 40 signature positions from positive samples of IBV. **B** Profile of 40 signature positions from negative samples of IBV. The *x*-axis represents the signature position in viral proteins. The *y*-axis represents the entropy value

The value 0.65 was set as the threshold for entropy screening, and 40 signature positions were achieved, as shown in Table 2. Each strain was represented by 40 amino acids to fulfill further machine learning (Fig. 3). The HA and NA proteins contained the most selected amino acid residues (14 for both), which suggested that HA and NA are the most important factors for human pathogenicity. HA is mainly involved in receptor binding, membrane fusion, and antigen recognition. Signature positions 115–231 are located in or near the region of receptor binding and the antigenic determinant group. The mutations at position 135 had the highest value of 1.06 (Table 2). As shown in Fig. 3, the deletion at HA161–161 should be noted because amino acid deletion can strongly affect protein function. NA influences the release of viral particles from the cell surface. The mutations in positions 120–392 are closed related to the enzyme activity of viral neuraminidase. NB is a viral protein with a short length and is related to virus replication. The role for two mutations at positions 21 and 99 should be further verified to understand the mechanism of pathogenicity. Although most signature positions shown in Fig. 3 were located in HA, NA, and NB proteins, the remaining eight mutations located at PB1, PA, NS1, or NEP proteins require additional attention during surveillance.

**Table 2:** Amino acid set for pathogenicity identification

Number	Protein	Position^a^	Entropy	Number	Protein	Position	Entropy
1	PB1	57	0.66	21	NA	49	0.82
2	PB1	752	0.68	22	NA	73	0.72
3	PA	352	0.73	23	NA	120	0.66
4	HA	47	0.70	24	NA	295	0.72
5	HA	74	0.69	25	NA	320	0.67
6	HA	115	0.67	26	NA	342	0.84
7	HA	128	0.94	27	NA	358	0.67
8	HA	132	0.70	28	NA	373	0.83
9	HA	135	1.06	29	NA	384	0.65
10	HA	145	0.69	30	NA	389	0.66
11	HA	149	0.67	31	NA	392	0.67
12	HA	161	0.99	32	NA	395	0.99
13	HA	162	0.70	33	NA	465	0.66
14	HA	173	0.65	34	NB	21	0.85
15	HA	200	0.68	35	NB	99	0.71
16	HA	228	0.72	36	NS1	111	0.85
17	HA	231	0.67	37	NS1	115	0.70
18	NP	9	0.66	38	NS1	120	0.67
19	NP	66	0.65	39	NS1	127	0.68
20	NA	45	0.66	40	NEP	88	0.71

*PB1* polymerase basic protein 1, *PA* polymerase acid protein, *HA* hemagglutinin, *NP* nucleoprotein, *NA* neuraminidase, *NB* glycoprotein NB, *NS1* nonstructural protein 1, *NEP* nuclear export protein

^a^B/Wisconsin/23/2019 (EPI_ISL_357982) as reference strain

### Optimal features with low dimension

**Fig. 4:**
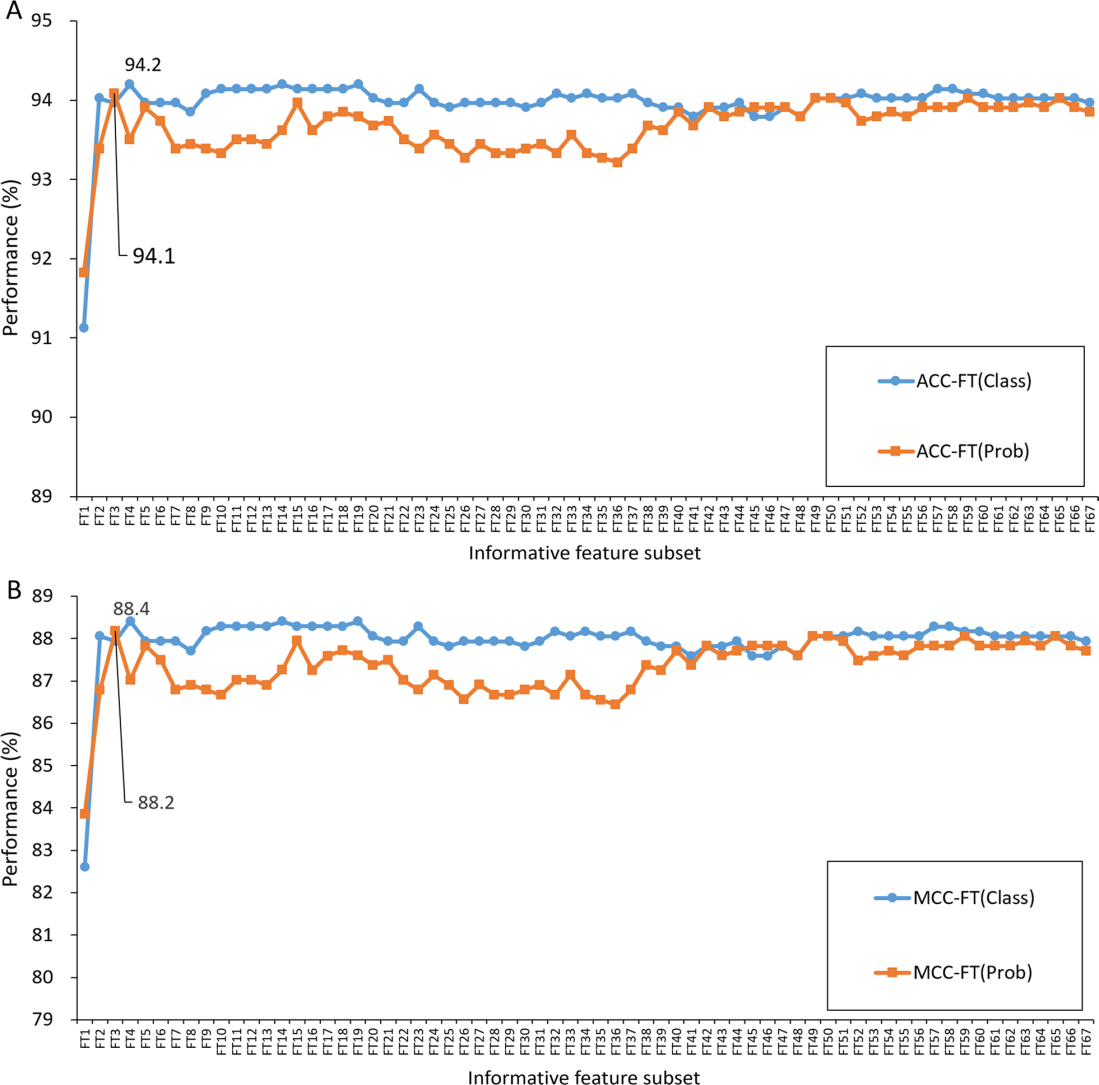
Optimization of informative features. **A** The SFS curves for the ACC of ‘class’ and ‘prob’ features. **B** The SFS curves for the MCC of ‘class’ and ‘prob’ features. The *x*-axis represents the incremental numbers of informative features. The *y*-axis represents the metric for the ACC and MCC. The ACC is marked in blue, while the MCC is marked in yellow

After the informative features were generated from the 67 RF predictors, the important scores for each feature were calculated by the mRMR algorithm. The SFS strategy was used to increase the ranked features one by one. The subset with best performance was considered to have the optimal features and was proposed to construct the final model for pathogenicity identification (Fig. 4). For the class features, a maximum ACC of 94.2% was achieved and coupled with the maximum MCC of 88.4%. The best performance was achieved when feature number 4 was selected, which suggests that the top four class features have the optimal representation of IBV. For the probabilistic features, the top three features produced the best model performance, with an ACC of 94.1% and MCC of 88.2%, which suggests that the top three probabilistic features have the optimal representation of IBV.

### Performance of the informative features

Two types of information features, the class label and probabilistic prediction, were received from the 67 RF predictors. As shown in Table 3, the features for class information slightly outperformed the features for probabilistic information. In terms of ACC and MCC, the performances based on class information were 94.0% and 87.9%, while those based on probabilistic information were 93.9% and 87.7%. The performance based on the optimal probabilistic features increased from 93.9 to 94.1% for ACC and from 87.7 to 88.2% for MCC. The performance based on optimal class features increased from 94.0 to 94.2% for ACC and from 87.9 to 88.4% for MCC. The performances of the optimal features were better than those of the original features.

**Table 3:** Performance of the informative features

Features	ACC	SE	SP	MCC	TP	TN	FP	FN
Class features	94.0	94.1	93.8	87.9	814	806	53	51
Probabilistic features	93.9	94.6	93.1	87.7	818	800	59	47
Optimal class features	94.2	95.0	93.4	88.4	822	802	57	43
Optimal probabilistic features	94.1	94.9	93.3	88.2	820	802	58	44

*SE* sensitivity, *SP* specificity, *ACC* accuracy, *MCC* Matthew’s correlation coefficient, *TP* true positive, *TN* true negative, *FP* false positive, *FN* false negative

### Comparison of informative features and amino acid features

In this paper, amino acid features were encoded from individual descriptors and input into the RF predictor to generate the informative features. To explore the power of the optimal subset of informative features, we compared the performance of the optimized informative features and the corresponding amino acid features. As shown in Table 4, there were differences in the performances of the optimal class feature and the amino acid features. The maximum ACC of 94.2% and maximum MCC of 88.4% were obtained from the optimal class feature, which were approximately 0.2–3% and 0.3–6% greater than those from amino acid features. It was notable that only four features were used for the optimal class feature, whereas OLP (k = 28) used 308 features, PC-PseAAC (λ = 6) used 27 features, GGAP (k = 5) used 441 features, and BIT20 (k = 12) used 240 features. The number for the optimal class feature was obviously lower than that for amino acid features.

**Table 4:** Performance of the optimal class features

Feature	ACC	SE	SP	MCC	TP	TN	FP	FN
Optimal class features	94.2	95.0	93.4	88.4	822	802	57	43
OLP (k = 28)	91.1	86.6	95.7	82.6	749	822	37	116
PC-PseAAC (λ = 5)	94.0	94.0	94.1	88.1	813	808	51	52
GGAP (g = 5)	93.6	92.7	94.4	87.1	802	811	48	63
BIT20 (k = 12)	91.0	86.2	95.7	82.3	746	822	37	119

*SE* sensitivity, *SP* specificity, *ACC* accuracy, *MCC* Matthew’s correlation coefficient, *TP* true positive, *TN* true negative, *FP* false positive, *FN* false negative, *PC-PseAAC* parallel correlation-based pseudo-amino-acid composition, *GGAP* the G-gap dipeptide composition, *BIT20* twenty-bit feature, *OLP* overlapping property feature

As shown in Table 5, there were also differences in the performances of the optimal probabilistic feature and corresponding amino acid features. The maximum ACC of 94.1% and maximum MCC of 88.2% were obtained from the optimal probabilistic feature, which were approximately 0.3–3% and 0.6–6% greater than those of amino acid features. It was also notable that only three features were used for the optimal probabilistic feature, whereas BIT21 (k = 32) used 672 features, BIT20 (k = 4) used 80 features, AAC used 20 features, and BIT21 (k = 4) used 84 features. The number for the optimal probabilistic feature was obviously lower than that for amino acid features.

**Table 5:** Performance of the optimal probabilistic features

Feature	ACC	SE	SP	MCC	TP	TN	FP	FN
Optimal probabilistic features	94.1	94.9	93.3	88.2	820	802	58	44
BIT21 (k = 32)	91.8	88.3	95.3	83.9	764	819	40	101
BIT20 (k = 4)	91.0	86.2	95.7	82.3	746	822	37	119
AAC	93.8	93.5	94.1	87.6	809	808	51	56
BIT21 (k = 4)	91.0	86.2	95.8	82.4	746	823	36	119

*SE* sensitivity, *SP* specificity, *ACC* accuracy, *MCC* Matthew’s correlation coefficient, *TP* true positive, *TN* true negative, *FP* false positive, *FN* false negative, *AAC* amino acid composition, *BIT20* twenty-bit feature, *BIT21* twenty-one-bit feature

### Comparison of SFS and ensemble strategy

The SFS strategy was used to search the optimal subset of informative features. To show the advantage of the SFS strategy, we compared the performances from the optimized informative features with those from two ensemble learning strategies (majority voting and probability averaging). The majority voting strategy considers the majority of class labels from the 67 RF models. The probability averaging strategy averages probabilistic values from the 67 RF models to perform classification. As shown in Table 6, the ACC for the SFS was approximately 0.7% greater than that for the majority voting strategies, while the MCC for the SFS was approximately 1.4% greater. The ACC for the SFS strategy was approximately 1% greater than that for the ensemble strategies, while the MCC for the SFS was approximately 2% greater. Both optimal features achieved better performance than the two ensemble methods.

**Table 6:** Performance of the SFS strategy

Learning strategies	ACC	SE	SP	MCC	TP	TN	FP	FN
Optimal class features	94.2	95.0	93.4	88.4	822	802	57	43
Optimal probabilistic features	94.1	94.9	93.3	88.2	820	802	58	44
Major voting	93.5	92.0	95.0	87.1	796	816	43	69
Probability averaging	93.0	90.9	95.2	86.2	786	818	41	79

*SFS* sequential forward search, *SE* sensitivity, *SP* specificity, *ACC* accuracy, *MCC* Matthew’s correlation coefficient, *TP* true positive, *TN* true negative, *FP* false positive, *FN* false negative

### Comparison of four classical classifiers

**Fig. 5:**
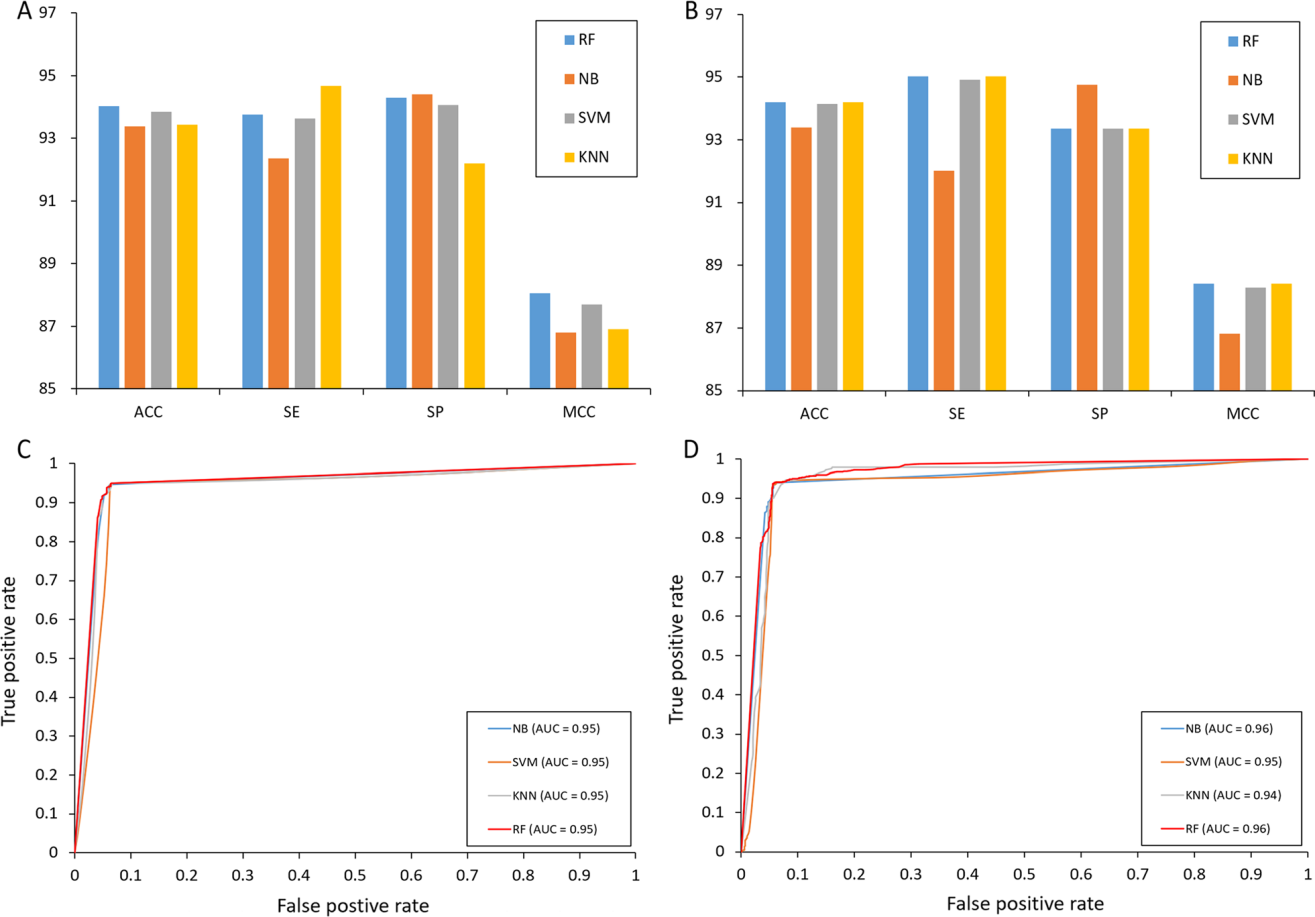
Comparison of four traditional classifiers. **A** Performances of the optimal ‘class’ features. **B** Performances of the optimal ‘prob’ features. **C** ROC curves of the optimal ‘class’ features. **D** ROC curves of the optimal ‘prob’ features

As mentioned above, the optimal features for class and probabilistic information had good performance. To use two types of the optimal features to identify pathogenicity of IBV, we compared the performances of RF, support vector machine (SVM), Naïve Bayes (NB), and K-nearest neighbor (KNN). All machine learning methods were evaluated with tenfold cross-validation. When the optimal class features were used, the RF method had better predictive performance than the NB and SVM methods and the same performance as the KNN method (Fig. 5A). The RF method obtained an ACC of 94.2% and MCC of 88.4%, which were approximately 1% and 1.4% greater than that of the NB method. The AUC for the RF method (0.95) was the same with those of the three other classifiers (Fig. 5C). When the optimal probabilistic feature was used, the RF method obtained an ACC of 94.0% and MCC of 88.1%, which were approximately 0.6% and 1.2% greater than that for the NB method (Fig. 5B). The AUC for the RF method (0.96) is the same as that for the NB method and is better than that for the SVM and KNN methods (Fig. 5D). According to the performances of four classical classifiers, the RF method was selected to treat the optimized informative features and construct the model for pathogenicity identification of IBV.

### Software implementation

An easy-to-use software freely accessible via https://github.com/kouzheng/BIVPred-FL was designed. The desired results can be easily achieved by the following steps: (1) Prepare the ‘FASTA’ file of amino acid sequences for IBV. Examples of formatted sequences can be found in the software directory. (2) Input the name of the query file, select the type of feature information, and set the confidence parameter as required. The predicted label for ‘P’ represents the phenotype of high pathogenicity, while ‘N’ means low pathogenicity. Amino acid features from the 67 individual descriptors were also delivered to facilitate further analysis.

## Discussion

In this study, we presented a method for pathogenicity identification of IBV to benefit public health [37]. The 40 signature positions were first achieved to represent each strain. After two types of informative features were generated from the 67 RF predictors, the mRMR feature ranking algorithm was used to select the optimal subset of informative features. The optimized informative features outperformed the original informative features and amino acid features from individual descriptors. The SFS strategy had better performance than two classical ensemble methods. Finally, the RF method was selected to treat the optimized informative features and construct the machine learning model to predict the phenotypes of IBV.

To reduce computing complexity, each strain was represented by 40 amino acids to fulfill further machine learning [22]. The HA and NA proteins contained the most selected amino acid residues (14 for both), which suggests that HA and NA are the most important factors for pathogenicity among humans. The role of two mutations at positions 21 and 99 should be further verified to understand the mechanism of pathogenicity. Although most signature positions are located in HA, NA, or NB proteins [15, 16, 38], eight mutations located in PB1, PA, NS1, or NEP proteins need extra attention during surveillance [14, 15, 17]. All signature positions were screened based on genome data of IBVs at a large scale, which will benefit the study of the pathogenicity mechanism [39].

Two types of informative features were generated by the RF predictors in this paper. Redundant and irrelevant features were filtered to improve the ability of IBV representation. Good performance was achieved with the use of four class features and three probabilistic features. The optimal subset with low dimensions reduced the complexity of computation. The optimal features about class information were achieved from four individual descriptors: OLP (k = 28), PC-PseAAC (λ = 6), GGAP (k = 5), and BIT20. The optimal features about probabilistic information were obtained from three individual descriptors: BIT21 (k = 32), BIT20 (k = 4), and AAC. The discrimination from different perspectives will benefit the accuracy and interpretability of pathogenicity [40].

Although IBV has not caused a pandemic, the risk of pathogenicity for a pandemic should also be considered [41]. IBV poses a serious threat to susceptible groups, such as children and adolescents, and can cause serious clinical complications. The monitoring of transmission and further research of pathogenicity mechanism should be increased. The method in this paper is a powerful tool for pathogenicity identification of IBVs at a large scale and can facilitate further study in the field of virology.

Although features from signature positions were used to construct the model, whole genomes and full-length proteins should be considered to increase the performance of the prediction model [26]. A mathematical algorithm should be designed for complex data of various models to identify pathogenicity [18, 42]. However, applying the algorithm to multimodal data will be a challenge. The main limitation of this study was that only amino acids in signature positions were encoded to build the prediction model, and the whole genome with clinical image data was not involved. Although the pathogenicity risk may be predicted in view of the pathogen, comprehensive judgment should be exercised to minimize pandemic risk [43].

## Conclusions

In this study, we presented a predictor for pathogenicity identification of IBV. The 40 signature positions were screened to represent each strain. Two types of informative features were generated from 67 RF models, and the mRMR algorithm was used to select the optimal subset. Based on the SFS strategy, the dimension of features about class information was optimized to four, with a maximum ACC of 94.2% and maximum MCC of 88.4%, and the dimension of features about probabilistic information was optimized to three, with a maximum ACC of 94.1% and maximum MCC of 88.2%. The optimal features outperformed the original informative features and amino acid features from individual descriptors. The SFS strategy had better performance than the two classical ensemble methods. The RF method was selected to predict the pathogenicity when optimal features were used as input. We believe that the method in this paper can serve as a powerful tool for pathogenicity identification of IBV and benefit public health.

## Supplementary Information

**Additional file 1.** The dataset for influenza B virus.

## Data Availability

After the registration for any application (https://www.gisaid.org/registration/register/), the public sequences of influenza viruses used in this paper can be downloaded from the GISAID EpiFlu database (http://platform.gisaid.org/epi3/frontend) under the database access agreement (https://platform.epicov.org/epi3/frontend#5aa0ce) and with the acknowledgment GISAID data contributors (https://www.gisaid.org/help/publish-with-data-from-gisaid/). We used the Python programming language to create an easy-to-use tool that implements our predictor and handle massive data, which is freely accessible via https://github.com/kouzheng/BIVPred-FL.

## Abbreviations

IBV: Influenza B virus
PB2: Polymerase basic protein 2
PB1: Polymerase basic protein 1
PA: Polymerase acid protein
HA: Hemagglutinin
NP: Nucleoprotein
NA: Neuraminidase
NB: Glycoprotein NB
M: Matrix protein
BM2: Matrix protein 2
NS1: Nonstructural protein 1
NEP: Nuclear export protein
AAC: Amino acid composition
PC-PseAAC: Parallel correlation-based pseudo-amino-acid composition
GGAP: The G-gap dipeptide composition
BIT20: Twenty-bit feature
BIT21: Twenty-one-bit feature
OLP: Overlapping property feature
mRMR: Minimum-redundancy maximum-relevancy
SFS: Sequential forward search
SE: Sensitivity
SP: Specificity
ACC: Accuracy
MCC: Mathew’s correlation coefficient
TP: True positive
TN: True negative
FP: False positive
FN: False negative
TPR: True positive rate
FPR: False positive rate
RF: Random forest
SVM: Support vector machine
NB: Naïve Bayes
KNN: K-nearest neighbor
ROC: Receiver operating characteristic curve
AUC: Area under the receiver operating characteristic curve
